# Evaluation of the Clinical Efficacy of a Novel Palmitoylethanolamide–*Equisetum arvense* Supplement for the Management of Chronic Pain: Findings from a Prospective Clinical Trial

**DOI:** 10.3390/medsci13030169

**Published:** 2025-09-03

**Authors:** Marco Invernizzi, Simone Mulè, Lorenzo Lippi, Rebecca Galla, Arianna Folli, Sara Ferrari, Domenico Tiso, Francesca Uberti

**Affiliations:** 1 Department of Health Sciences, University of Piemonte Orientale (UPO), 28100 Novara, Italy; marco.invernizzi@med.uniupo.it (M.I.); arianna.folli23@gmail.com (A.F.); 2 Translational Medicine, Dipartimento Attività Integrate Ricerca e Innovazione (DAIRI), Azienda Ospedaliero-Universitaria (AOU) SS, Antonio e Biagio e Cesare Arrigo, 15121 Alessandria, Italy; lorenzo.lippi@ospedale.al.it; 3 Department for Sustainable Development and Ecological Transition, University of Piemonte Orientale (UPO), 13100 Vercelli, Italy; simone.mule@uniupo.it (S.M.); sara.ferrari@uniupo.it (S.F.); 4 Noivita Srls, Spin Off, University of Piemonte Orientale (UPO), Strada Privata Curti 7, 28100 Novara, Italy; rebecca.galla@uniupo.it; 5 Department of Clinical Nutrition, “Villa Maria” Hospital, 47921 Rimini, Italy; dottortiso@gmail.com

**Keywords:** oral supplement, neuroinflammation modulation, neuroprotective supplementation, pain-related disability, analgesic effect, chronic pain

## Abstract

**Background**: Chronic pain represents a major therapeutic challenge due to the limited efficacy and tolerability of conventional pharmacological treatments. *Equisetum arvense* L., a medicinal plant with potent antioxidant properties, and palmitoylethanolamide (PEA), an endogenous fatty acid amide with well-established anti-inflammatory and analgesic effects, are increasingly recognised as promising nutraceutical agents. **Methods**: This prospective, single-centre clinical trial aimed to evaluate the efficacy and safety of a novel oral supplement (Assonal^®^PEA) combining 600 mg of PEA and 300 mg of *Equisetum arvense* L. in improving the reduction of pain and quality of life in patients with chronic pain, also obtaining information on the patient’s state of satisfaction after the treatment. Fifty patients suffering from chronic pain (low back pain and radiculopathy) for two months were enrolled and received the supplement over eight weeks in a tapered regimen (two tablets daily for two weeks, followed by one tablet daily). **Results**: Clinical outcomes were evaluated using validated instruments, including the Numeric Pain Rating Scale (NPRS), Verbal Rating Scale (VRS), Short-Form McGill Pain Questionnaire (SF-MPQ), Global Perceived Effect (GPE), and EuroQol-5D-5L. Results showed a significant decrease in pain intensity (NPRS: −3.8 points; VRS: −2.1 points; *p* < 0.0001), along with meaningful improvements in patient-perceived benefit, pain descriptors, and quality of life (EQ-5D-5L: +35%; *p* < 0.0001). **Conclusions**: These findings endorse the use of this novel PEA–*Equisetum arvense* formulation as a safe, well-tolerated, and potentially effective supplementary intervention for managing chronic pain. No adverse events were reported, and the overall response rate reached 94%.

## 1. Introduction

In contrast to acute pain, which is a physiological warning sign, chronic pain lasts longer than anticipated—often months or even years—and places a significant strain on both individuals and healthcare systems [1]. The International Association for the Study of Pain (IASP) defines chronic pain as “an unpleasant sensory and emotional experience associated with or resemblance to that associated with a real or potential tissue year of pain” [2]. Chronic pain is a pervasive and debilitating condition that affects millions of individuals worldwide with an estimated 20–30% of the adult population, significantly impairing their functional abilities, emotional well-being, sleep quality, and overall quality of life [3]. In Europe, epidemiological surveys report a prevalence of around 19%, with the majority of cases being of musculoskeletal origin, particularly low back and neck pain, osteoarthritis, and other degenerative joint conditions [4] These conditions often have a multifactorial origin, including mechanical overload, degenerative changes, postural alterations, and, in some cases, neuropathic components. Conventional pharmacological treatments for chronic pain primarily involve nonsteroidal anti-inflammatory drugs (NSAIDs), opioids, and antidepressants [5]. While these medications provide symptomatic relief, they are often associated with severe side effects, including gastrointestinal irritation, renal complications, increased cardiovascular risks, and, in the case of opioids, dependence and addiction [6]. Given these limitations, there is an urgent need to explore alternative therapies that are both effective and safe for long-term pain management.

In this scenario, dietary supplements have emerged as a promising adjunctive therapy for managing chronic pain, providing a natural and potentially safer alternative to conventional pharmacological treatments. Several nutraceuticals, including various herbal and botanical extracts such as *Boswellia serrata*, *Zingiber officinale* and *Harpagophytum procumbens*, have been investigated for their potential to improve chronic pain management [7,8,9]. These supplements typically contain bioactive compounds such as beta-carotene and other carotenoids, zingiberene, boswellic acids, harpagoside with anti-inflammatory and antioxidant properties, which may offer effective alternatives to traditional medications to manage chronic pain [7,8,9,10].

In recent years, the potential of palmitoylethanolamide (PEA) has been highlighted as a promising non-pharmacological option for chronic pain relief [11,12,13]. PEA is an endogenous fatty acid amide known for its anti-inflammatory, neuroprotective, and analgesic properties [14]. It exerts its effects by modulating the endocannabinoid system, reducing mast cell activation, and inhibiting the release of pro-inflammatory mediators such as tumour necrosis factor-alpha (TNF-α) and interleukin-1 beta (IL-1β) [15,16]. These mechanisms reduce pain perception and neuroinflammation, making PEA a valuable candidate for managing conditions such as neuropathic pain, fibromyalgia, and osteoarthritis [17]. In addition to PEA, the medicinal plant *Equisetum arvense* L. (horsetail) has gained attention for its anti-inflammatory, antioxidant, and regenerative properties [13,18]. Traditionally used in herbal medicine, *Equisetum arvense* L. contains bioactive compounds such as flavonoids, silica, and phenolic acids, which may help reduce oxidative stress and inflammation, complementing PEA’s analgesic effect in the treatment of chronic pain in humans [19]. The synergistic action of these two compounds could offer a novel and effective approach to managing chronic pain while minimising the adverse effects associated with conventional treatments. Although both compounds have individually documented analgesic effects [20,21,22,23], no previous study has investigated the clinical efficacy of their combination in chronic musculoskeletal pain, neither has any study evaluated their integrated effect on pain intensity, quality of life in a human population with persistent symptoms for more than 3 months. Therefore, this study aims to evaluate the clinical efficacy of Assonal^®^PEA (Agave Group srl, Bologna, Italy), a novel formulation combining PEA (600 mg) and *Equisetum arvense* L. (300 mg) in patients with chronic pain. Specifically, this study aimed to evaluate the clinical efficacy of Assonal^®^PEA in reducing pain, improving quality of life, and assessing patient satisfaction following treatment in patients with lumbar musculoskeletal pain. The study hypothesised that combined administration of PEA and *Equisetum arvense* L. would lead to greater improvement in pain control, physical function during normal quotidian life activities, and overall quality of life compared to baseline, with a favourable safety and tolerability profile. Our study provides for the first evidence in humans of the potential benefit of this nutraceutical combination in a chronic pain setting.

## 2. Materials and Methods

### 2.1. Study Design and Population

**Figure 1:**
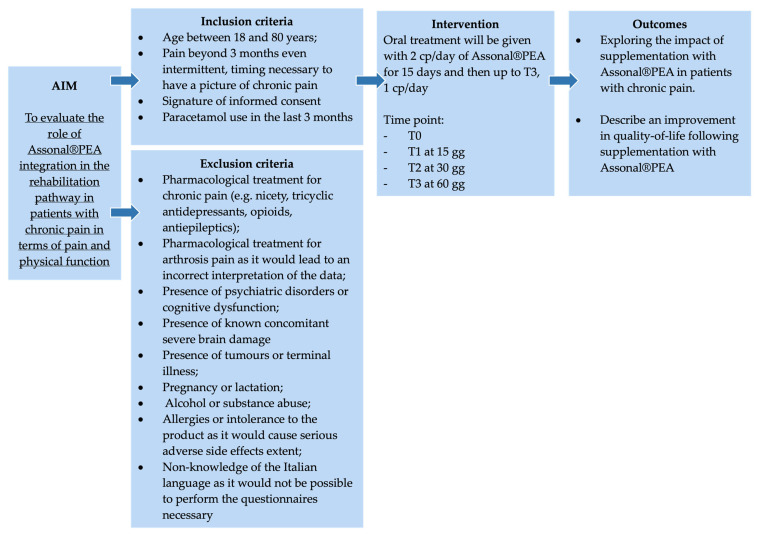
The summary scheme of the clinical study.

This single-center, non-controlled clinical study was conducted at “Azienda Ospedaliero Universitaria” (A.O.U.) Saints (SS.) Antonio and Biagio and Cesare Arrigo Hospital in Alessandria (Italy) starting from the date of study authorisation (Practice N° CE148/2024 of the Territorial Ethics Committee [TEC] Interagency A.O.U. Maggiore della Carità in Novara, Italy) (Figure 1). The study was conducted in compliance with current Italian and European legislation on clinical trials and the protection of personal data (Decree no. 196/2003 and 679/2016—GDPR), and in compliance with the Declaration of Helsinki for the entire duration of the study.

From the date of study authorisation, all patients refered to the A.O.U. SS. Antonio e Biagio and Cesare Arrigo of Alessandria, with a diagnosis of chronic pain in terms of low back pain and radiculopathy, were evaluated to verify that they fit the study inclusion and exclusion criteria described in the next section. An informed consent document is provided to all subjects who meet the above parameters. The delivery is made by the principal investigator or a specially identified and professionally registered collaborator to explain to the patients all the characteristics and evaluations included in the protocol and will answer any questions. For each subject who signed the informed consent document, an informational letter was sent to the treating physician to proceed with the evaluations under this project.

The present study was conducted as a before-after clinical trial. This methodology involves the evaluation of each participant at two (or more) time points: “before” (T0) and “after” (T1, T2, T3) the intervention under consideration. In this design, each subject acts as their own internal control, allowing individual conditions before and after exposure to the treatment to be directly compared. This approach is particularly useful when an external control group is not available or is not ethically or logistically feasible [24]. Although the absence of a comparison with a placebo or otherwise treated group represents a limit in terms of causal inference, the before-after study reduces interindividual variability and allows for the observation of significant changes associated with the intervention, considering the specific initial conditions of each participant. In addition, this study design allows for monitoring the evolution of clinical parameters over time and to identify response patterns, even without randomisation [25].

The study enrolled patients diagnosed with chronic pain, defined as pain persisting for more than three months. In this context, patients with chronic pain are defined as patients who complain of persistent pain sensation beyond 3 months. Indeed, chronic pain is generally determined by its duration (persistence of pain beyond 3 months) and/or the fact that it persists despite treatment [26]. Chronic pain differs from neuropathic pain in that, compared with the former, which is indicative of duration, the latter refers to pain caused by an injury or disease of the somatosensory nervous system. Furthermore, an injury or disease is considered peripheral when it affects the peripheral system, and it is considered central when the central nervous system is affected [27].

The study was divided into four phases according to different evaluation time points:

- T0 (enrolment of the subject in the study): After explaining the treatment administration dosage (two tablets per day of Assonal^®^PEA) and submitting the questionnaires for the first time, this group becomes the control group for the next phases (Before).
- T1: After 15 days of oral treatment (two tablets a day of Assonal^®^PEA), evaluations with questionnaire administration will follow. At this stage, patients will adjust the treatment dosage to one tablet per day.
- T2: After one month of oral treatment (one tablet per day of Assonal^®^PEA), evaluations with questionnaire administration will follow.
- T3: After two months of treatment taken orally (one tablet per day of Assonal^®^PEA), final evaluations were conducted with the administration of the questionnaires.

The patient is scheduled to be visited at the A.O.U at each assessment time point. SS. Antonio e Biagio and Cesare Arrigo of Alessandria for assessments, which are administered using questionnaires with the approval of the attending physician.

### 2.2. Eligibility Criteria of the Study Population

#### 2.2.1. Inclusion Criteria

- Patients between 18 and 80 years of age.
- Pain beyond 3 months, even intermittent, timing is necessary to have a pattern of chronic pain.
- Signature of informed consent.
- Paracetamol use in the last 3 months.

#### 2.2.2. Exclusion Criteria

- Drug treatment for chronic pain (e.g., acetyl-L-carnitine, tricyclic antidepressants, opioids, antiepileptics) would lead to an incorrect interpretation of data.
- Pharmacological treatment for arthritis pain would lead to an incorrect interpretation of the data.
- Psychiatric disorders or cognitive dysfunction in patients with these disorders could result in the completion of questionnaires that are not useful for the final assessment.
- Concomitant severe brain damage.
- Tumours or terminal illnesses.
- Pregnancy or lactation.
- Alcohol or substance abuse.
- Allergies or intolerance to the product as it would cause serious adverse side effects.
- Non-knowledge of the Italian language as it would not be possible to perform the questionnaires necessary

It was ensured that all patients reported at least moderate pain, operationally defined as NPRS ≥ 5 or VRS ≥ “Moderate pain”, so ≥5. This approach is in line with clinical practice and maximises the ability to detect significant improvements while avoiding floor effects, as also observed in similar clinical trials where an NPRS/VRS or/VAS score ≥ 5 was used as a reference [28]. Patients were asked to refrain from using any other dietary supplements, herbal extracts, or natural products with known or potential analgesic, anti-inflammatory, or neuromodulatory activity during the study period. Compliance with this recommendation was verified during screening and at each follow-up visit. If patients reported taking NSAIDs, pain relievers or anti-inflammatories during the study period, they would be automatically excluded, marked as “dropouts”, and considered during data analysis.

### 2.3. Product Under Investigation

The product evaluated in the following protocol is a new formulation containing 600 mg of PEA and 300 mg of *Equisetum arvense* L. (Assonal^®^PEA) per tablet to reduce chronic pain symptoms.

Precisely, Assonal^®^PEA, with its unique formulation based on EQUIPEA^®^, contributes to the body’s homeostasis and is useful in all cases of insufficient dietary intake or increased need for these nutrients [13]. PEA, an endogenous lipid mediator, is present in the body and belongs to the ALIAmide family. The term ALIAmide stands for ‘Autacoid Local Injury Antagonist’ and was coined by Rita Levi Montalcini to describe a group of endogenous bioactive molecules involved in the homeostasis of inflammatory processes [29]. PEA is a substance widely present in various tissues, including those belonging to the nervous system, and is synthesised by the body when needed. *Equisetum arvense* L., an herbaceous perennial plant, is very rich in minerals and nutrients. *Equisetum arvense* L. also contains important active ingredients: flavonoids, saponins and phytosterols, optimal for maintaining peripheral nerve health [30]. As the manufacturer indicates, Assonal^®^PEA is stored in a cool, dry place, avoiding exposure to localised heat sources and sunlight and keeping it away from moisture. Additionally, the tablets were kept inside the blister pack until the time of intake [31].

The enrolled patient was advised to take 1 to 2 tablets daily at any time, preferably with a sip of water, as directed by the pharmaceutical company [31]. In detail, starting with the distribution of Assonal^®^PEA (T0) by the principal investigator, patients took 2 tablets per day (1 in the morning and 1 in the evening) for the first 15 days of treatment (T1). Treatment continued with 1 tablet per day until the end of the clinical study (T3).

### 2.4. Endpoints

#### 2.4.1. Primary Endpoint

The primary endpoint was the reduction of perceived pain, which was measured using the NPRS and VRS [32] at T0, T1, T2, and T3. The NPRS (or NRS scale) is a one-dimensional 11-point scale that assesses the intensity of pain in adults [33], including chronic pain conditions due to rheumatic diseases [34,35]. The scale is composed of a horizontal line, with an interval ranging from 0 to 10, corresponding to “no pain” and “worst imaginable pain”, respectively. The NPRS scale can be easily administered both verbally (for example, also by telephone) and graphically [36]. The compilation time is less than a minute and does not require any visual-motor coordination, unlike the VAS scale. There are no barriers to translating the NPRS scale into other cultures or languages. Test-retest reliability was good but higher among literate patients (r = 0.96) than illiterate patients (r = 0.95). The advantages of NRS are simplicity, reproducibility, ease of comprehension, and sensitivity to small changes in pain [32].

#### 2.4.2. Secondary Endpoints

The secondary endpoints were:

- The measurement of patient perception versus drug treatment, which will be measured with the GPE, a scale that assesses patient perception using a 7-point Likert scale [37]. This is a subjective assessment in which the patient indicates how much they feel improved or worsened compared to before treatment (1: Very much worse; 2: Moderately worsened; 3: Slightly improved; 4: No change; 5: Slightly improved; 6: Moderately improved; 7: Much improved). This scale is widely used in clinical trials and clinical practice to assess the effectiveness of therapeutic interventions, particularly in musculoskeletal and rehabilitation settings.
- The quantification and description of perceived pain will be assessed using the SF-MPQ [38]. It is a self-assessment questionnaire that allows you to have an accurate description of the quality and intensity of the pain that the subject is experiencing. There are 78 items divided into 20 subgroups that refer to 4 main categories: the sensory class, the affective class, the valuative class and the miscellaneous class [39]. The SF-MPQ also includes the Pain Intensity Index (PPI) of the standard MPQ and a visual analogue scale (VAS).
- Measuring quality of life, described with the EURO-QoL-5D-5L or EQ-5D-5L. The instrument consists of: a 5-dimensional descriptive system (mobility, self-care ability, habitual activities, pain/discomfort, and anxiety/depression), each of which involves a three-point rating scale (no problem = 1 point; some problem = 2 points; persistent problem = 3 points); and a VAS that requires the subject to answer the question, “How healthy do you feel today?” A score of 0 corresponds to the worst health status ever, while 100 equals the best perceived health level. It is possible to combine the scores of the two sections and determine an overall score [40].

### 2.5. Statistical Analysis

The sample size calculation was performed using G*Power3 software, focusing on the primary endpoint. Considering a delta of pain improvement after treatment with Assonal^®^PEA of a mean value of 2.4 points, consistent with evidence from other studies [41]. There is a need to enrol at least 50 participants. Considering a possible dropout of 25% (α = 0.05 and β = 0.20). Statistical analysis was carried out using GraphPad Prism 10.2.3 (GraphPad Software, Inc., San Diego, CA, USA). Although 50 participants were initially enrolled, one subject was lost at T2, which was considered during data analysis as a dropout. All longitudinal analyses were then performed on the remaining 49 participants with complete datasets at all time points.

Data were reported as mean values ± standard deviations. Friedmann’s sum test performed statistical comparisons at different time points (T1, T2 and T3) to assess differences from the start of treatments (T0). Pain scores were analysed by effect over time, and treatment was done using the two-way ANOVA method to evaluate whether treatment with Assonal^®^PEA improved pain management compared with the initial study condition. The level of significance is set with a threshold level of 0.05.

## 3. Results

**Figure 2:**
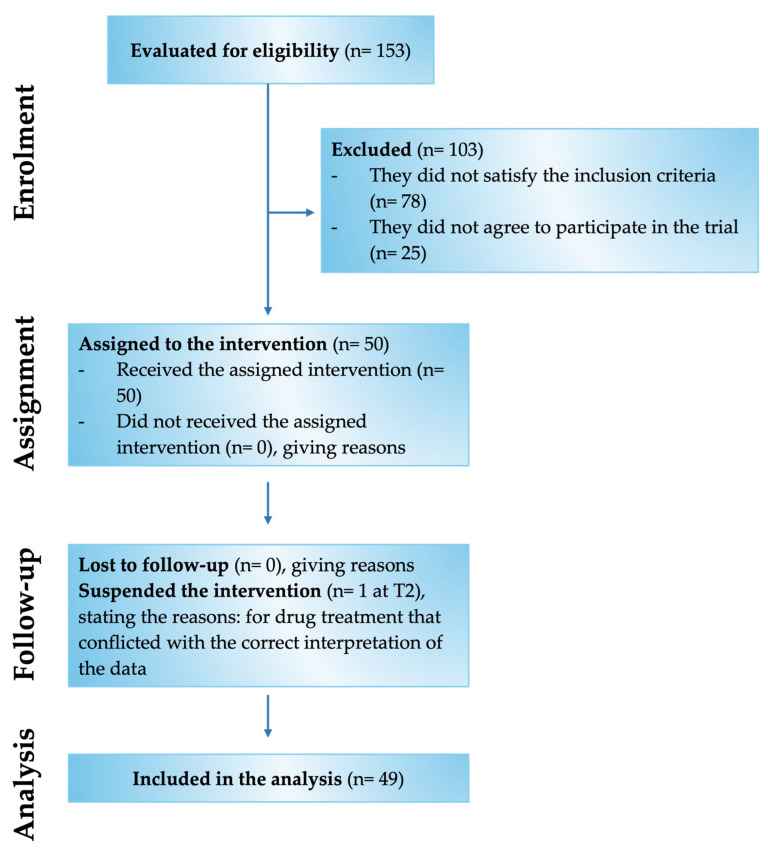
Flow Chart of the various phases of the Assonal^®^PEA clinical study.

Of the 153 patients assessed for eligibility, 50 met the inclusion criteria and were enrolled in the study (Figure 2). Specifically, the study involved 28 male and 22 female patients with chronic pain for more than 3 months and compatible with the selected inclusion and exclusion criteria (Table 1). In detail, the patients examined had chronic pain mainly related to low back pain and radiculopathy. The mean age was 67.3 ± 10.9 y/o, of which 31 patients were aged over 60 and 19 patients were aged under 60.

Of these 50 enrolled patients, 49 completed the study, while only 1 patient was recorded as a dropout at time point 2 (T2). Of the 49 patients indicated above, 46 were responsive to treatment from a beneficial point of view on chronic pain. In comparison, 3 patients reported no improvement and worsening by being non-responsive to Assonal^®^PEA.

### 3.1. Effects of Assonal^®^PEA on Pain Intensity

**Figure 3:**
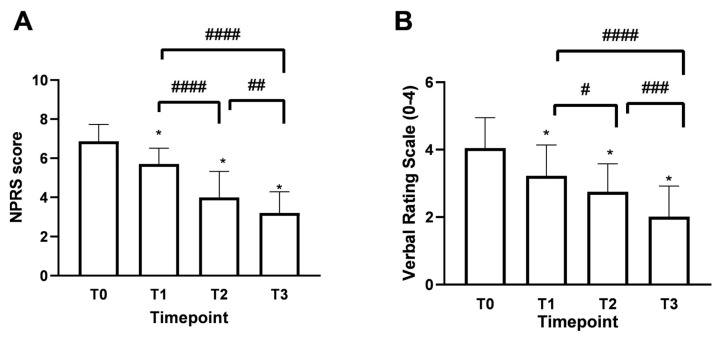
Results obtained on the study’s primary endpoint after treatment with Assonal^®^PEA from T0 to T3 following the recruitment protocol. In (**A**), the results were obtained in terms of NPRS, and (**B**), in terms of VRS. The data are reported as mean ± SD of the self-reported scores after administration of the questionnaires by the 50 patients at all examination time points. In (**A**) * *p* < 0.0001 vs. T0; #### T3 *p* < 0.0001 vs. T1 and T2 vs. T1; ## T3 *p* = 0.0012 vs. T2. In (**B**) * *p* < 0.0001 vs. T0; #### T3 *p* < 0.0001 vs. T1; # T2 *p* = 0.048 vs. T1; ### T3 *p* = 0.0004 vs. T2.

Patients over 60 years of age, accounting for 61% of the total enrolled patients, evidenced a chronic pain rating of 6.92 ± 0.90, which is equivalent to very acute pain. At the same time, the remaining 33% indicated more moderate pain with a Numerical Pain Rating Scale (NPRS) value of around 6.40 ± 0.83. As shown in Figure 3A, the mean NPRS score at baseline (timepoint 0, T0) was 6.8, indicating a strong perception of pain with acute intensity. As early as 15 days of treatment (timepoint 1, T1), a reduction of 1.1 points was observed, bringing the score to 5.75 ± 0.78 with moderate-type pain intensity. In more detail, after 15 days of treatment with Assonal^®^PEA (T1), the most pronounced beneficial effect of pain reduction occurred in patients older than 60. In this group of patients, the NPRS value was reduced by 1.2 points (21% reduction); on the contrary, in the age group below 60 years, the effect at T1 was more dampened by a reduction of 0.6 points (11%). Continuing with the treatment after one month (T2) and two months (T3), with a dose reduction from 2 to 1 tablet per day starting at the end of T1, the NPRS score decreased further, reaching a score of 3.0 ± 1.15 (77% reduction) at T3 (*p* < 0.0001) associated with patient-reported mild pain perception. In the second part of the study, the beneficial effect of Assonal^®^PEA reached a maximum reduction in pain intensity in patients younger than 60 years, reducing the NPRS value by 3 (1.3-fold) and 3.4 (1.7-fold) points at T2 and T3, respectively, compared to T0. At the same time, in the older group examined, the effect of Assonal^®^PEA was less marked but still effective by reducing the NPRS value by 1.8 (77%) and 2.5 (1.15-fold) points at T2 and T3, respectively. At the end of the research period (T3), averages for patients in both age groups who received Assonal^®^PEA treatment showed that their NPRS score decreased by 3.8 points (1.15 times) as compared to T0. The results were also broadly confirmed by the Verbal Rating Scale (VRS, Figure 3B), which recorded statistically significant differences (*p* < 0.0001) following the one month of intake (T2) and after the two months of intake (T3) of Assonal^®^PEA even by reducing the dose from 2 to 1 tablet, reaching a VRS score value of 2 points (83% reduction) at T3 compared to T0 in which a value of 4.1 ± 1.12 reported by patients.

### 3.2. Effects of Assonal^®^PEA in the Secondary Endpoints

**Figure 4:**
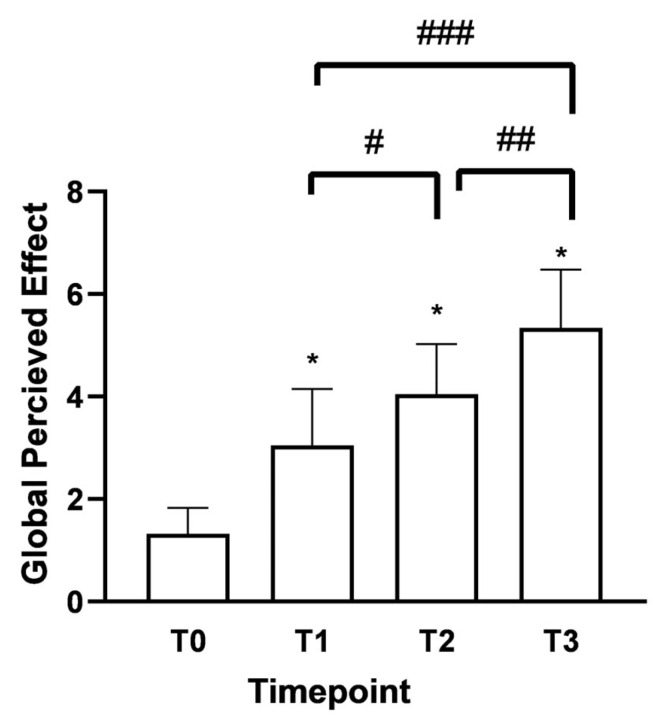
Results were obtained regarding the degree of satisfaction with the treatment received/performed (GPE score) following oral intake of Assonal^®^PEA from T0 to T3, following the examination protocol. The data are reported as the mean ± SD of the self-reported scores obtained after administering the questionnaire to 50 patients at all examination time points. * *p* < 0.0001 vs. T0; # T2 *p* < 0.0001 vs. T1; ## T3 *p* < 0.0001 vs. T2; ### T3 *p* < 0.0001 vs. T1.

As shown in Figure 4, the Global Perceived Effect (GPE) score at T0 was 1.3 (indicating the presence of the pathological problem). After 15 days (T1) of Assonal^®^PEA administration, it rose to 3.05 with significant differences between the time points. In both age groups under consideration, there was the same trend of improvement in the perception of treatment received with a phase of neutrality to treatment, i.e., “it did not improve or worsen problem” according to the GPE scale; 4.15 ± 0.98 (32% increase) for over 60 and 4 ± 0.94 (42% increase) for under 60 at T2 compared to T1. The most considerable statistically significant difference compared to T0 (*p* < 0.0001) was recorded following the month of intake (T2). After two months of intake, (T3) of Assonal^®^PEA also reduced the dose from 2 to 1 tablet, reaching a value at T3 of 5.3 ± 1.06 (76% increase), indicating a partial improvement of the problem, as reported by patients.

**Figure 5:**
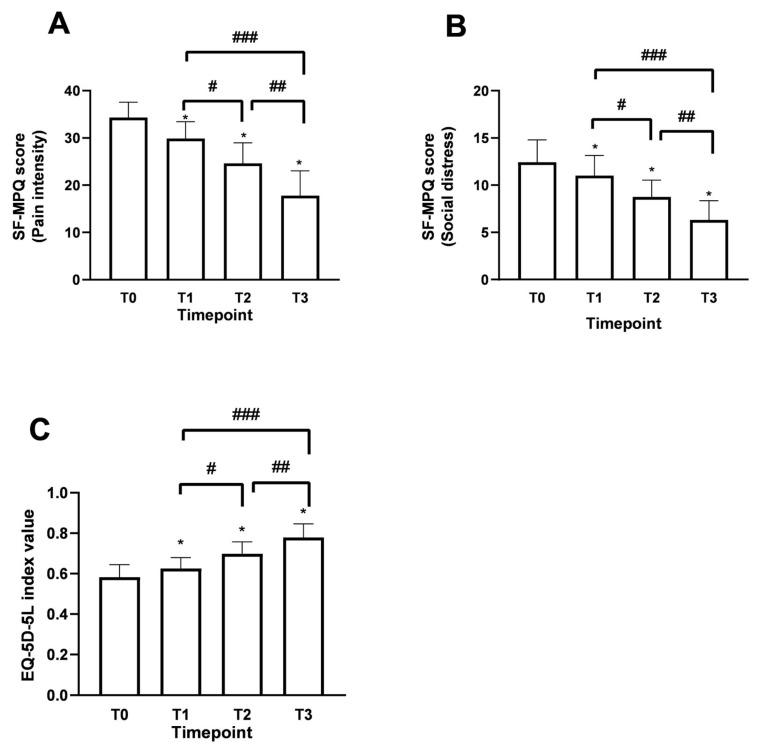
Results obtained in terms of improvement in the described pain state and quality of life following oral intake of Assonal^®^PEA from T0 to T3 according to the examination protocol. In (**A**,**B**), the results regarding SF-MPQ (in terms of pain intensity and social distress, respectively) were obtained, and in (**C**), the results relating to EQ-5D-5L were obtained. The data are reported as the mean ± SD of the self-reported scores obtained after administering the questionnaire to 50 patients at all examination time points. (**A**) * *p* < 0.0001 vs. T0; # T2p = 0.0002 vs. T1; ## T3 *p* < 0.0001 vs. T2; ### T3 *p* < 0.0001 vs. T1; (**B**) * *p* < 0.0001 vs. T0; # T2 *p* = 0.0002 vs. T1; ## T3 *p* < 0.0001 vs. T2; ### T3 *p* < 0.0001 vs. T1; (**C**) * *p* < 0.0001 vs. T0; # T2 *p* < 0.0001 vs. T1.; ## T3 *p* < 0.0001 vs. T2; ### T3 *p* < 0.0001 vs. T1.

Using the Short Form McGill Pain Questionnaire (SF-MPQ) allowed us to identify fluctuations in patient perception compared to the description of perceived pain since the beginning of the supplementation, reinforcing the data obtained for the study’s primary endpoint. The SF-MPQ scale allowed for characterising fluctuations in the perceived pain status by analysing various parameters (20), descriptively divided into 4 main groups: sensory (1–10), affective (11–15), evaluative (16), miscellaneous (17–20). Similar claims were found for both age groups examined during the various time points. Starting from T0, patients aged over 60 years have had an average score of 26.3 ± 2.52 in the sensory category, which describes pain in terms of pressure, localisation and time. In the affective section, which describes pain in psychological and endurance terms, an average value of 9 ± 1.77 was calculated. In the evaluative section, which describes pain as intense, problematic, or unbearable, the average value was 4 ± 0.50, correlating with high intensity. Finally, in the miscellaneous section, which describes pain in various disabling aspects, an average value of 7.5 ± 1.45 was obtained. Similar results were also recorded in the under-60 age group. Therefore, as shown in Figure 5A (pain intensity: items 1–11), the data, in terms of SF-MPQ score, indicate an average value at T0 reported by patients of 34.3 ± 3.23, an indicator of a moderate perception and description of pain suggested by the patient. Following the intake of Assonal^®^PEA, a statistically significant reduction (*p* < 0.0001) was achieved after the month of intake (T2) and after two months of intake (T3) of Assonal^®^PEA, also reducing the dose from 2 to 1 tablet, reaching an SF-MPQ core value at T3 equal to 17.7 ± 5.11 and specifically, promoting significant effects in terms of pain reduction that affects the psychological aspect of the patient (Figure 5B), in both age groups. The net reduction from T0 was about 16.6 points (94% reduction; *p* < 0.0001). At the same time, it was possible to define during the treatment period to correlate the intensity of pain with the surrounding environment, allowing us to study the role of Assonal^®^PEA in chronic pain that can become disabling to the individual’s social life (Figure 5B). Consistent with the data obtained in Figure 5A, compared with the baseline condition, with a total score value of about 12.4 ± 2.37, Assonal^®^PEA helped to improve the impact of pain on normal and social activities of daily living (Figure 5B) by reaching a value at T3 of 6.3 ± 2.02, reducing the score by 6.1 points (97% reduction; *p* < 0.0001).

Data consistent with the previous were also obtained after evaluating the improvement in quality of life following the treatment under consideration. Using the European Quality of Life Five Dimension Five Level Scale (EQ-5D-5L), it was possible to identify clear and precise fluctuations in the quality improvement perceived by the patient. As can be seen from the data shown in Figure 5C, in terms of EQ-5D-5L, at T0, a value equal to 0.58 ± 0.13 was recorded, which is assumed to refer to a poor quality of life perceived by the patient suffering from chronic pain. Following the intake of Assonal^®^PEA, the score showed a statistically significant difference (*p* < 0.0001) after the first month of intake (T2). After two months of intake, (T3) of Assonal^®^PEA even reducing the dose from 2 to 1 tablet, reaching a score value at T3 of 0.77 ± 0.15 (35% increase; *p* < 0.0001), indicating an improvement in quality of life compared to the starting condition, in line with previous data on the perception of chronic pain and its intensity.

## 4. Discussion

Nutraceuticals, including PEA and *Equisetum arvense* L., represent a promising approach in the treatment of chronic pain intensity. They encompass a wide range of dietary products ranging from supplements and isolated nutrients to functional foods and herbal preparations that provide health benefits and may play a role in disease prevention or therapy [42,43]. Chronic pain affects millions of individuals worldwide and remains a major health challenge due to its disabling nature and the limited efficacy of current treatment options [44,45,46,47]. Several clinical trials have reported that PEA is effective in reducing pain intensity in various chronic pain conditions, such as neuropathic pain, low back pain, and osteoarticular disorders, with an excellent safety profile and minimal side effects [48,49]. PEA is an endogenous fatty acid amid that has demonstrated significant analgesic and anti-inflammatory properties in both preclinical and clinical studies. Its mechanism of action is primarily related to the modulation of mast cells, microglia, and other non-neuronal cells involved in neuroinflammation and chronic pain. PEA acts through multiple pathways, including the activation of the peroxisome proliferator-activated receptor alpha (PPAR-α), leading to the downregulation of pro-inflammatory mediators and the restoration of neuroimmune balance [17,50,51]. Our present study demonstrated that PEA supplementation significantly reduced pain levels in individuals with chronic musculoskeletal pain, confirming its potential as an effective analgesic agent. In this context, it has been established that administering exogenous PEA effectively mitigates neuroinflammation at the cellular level only when in micronised (mPEA, range 2–10 μm) or ultra-micronised (umPEA, range 0.8–6 μm) forms [52,53,54], while the native form, indicates how naïve PEA, suffers from poor absorption due to its larger particle size (2000 μm), which greatly limits its distribution and bioavailability, resulting in a minimal biological effect [28]. Indeed, the mPEA and umPEA have been shown to promote greater bioavailability, allowing the molecule to more effectively reach the target tissue [55,56]. This is in line with the data obtained in terms of reducing the intensity of pain by improving the individual’s health condition during this study, as Assonal^®^PEA contains a form of PEA with a specific cut-off of 80 mesh (PEA 80mesh), or approximately 177 μm. The analgesic effect observed in our study is consistent with previous findings and can be explained by PEA’s biological mechanisms. PEA exerts its action primarily through the activation of PPAR-α, which downregulates the expression of pro-inflammatory cytokines such as TNF-α, IL-1β, and interleukin-6 (IL-6) [57]. Furthermore, PEA inhibits the activation of mast cells and microglia, key players in neuroinflammation and pain sensitisation [58]. This mechanism supports the reduction in peripheral and central sensitisation, contributing to the relief of chronic musculoskeletal pain [59]. In addition, such as after Assonal^®^PEA treatment, numerous clinical studies have demonstrated the formulations’ time-dependent effectiveness in treating chronic pain [48,60]. Short-term prospective data on oral umPEA therapy for traumatic pain or chronic diabetes were provided in a study. Pregabalin, gabapentin, or tramadol had been used to treat all the participants in the past, but the results showed only modest relief and considerable lingering pain. The VAS score dramatically dropped after 40 days of treatment, first after 10 days and then again at the end of treatment. The quality of life, as assessed by the EQ-5D scale and the Neuropathic Pain Symptom Inventory (NPSI) score for paresthesia and dysesthesia, both showed notable improvements [55]. The clinical results obtained following the oral intake of Assonal^®^PEA made it possible to promote a significant transposition with what was previously obtained on in vitro 3D models validated in the literature [54]; also selecting an optimal dosage. Indeed, PEA 80mesh demonstrated optimal absorption kinetics in a 3D in vitro model of the intestine compared to forms such as mPEA and umPEA, as well as good anti-inflammatory and peripheral nerve health maintenance results [54]. PEA 80mesh allowed not only a reduction in the inflammatory context by modulating not only TNF-α which, related to inflammation, contributes to raising awareness of nociceptors, pain and neuronal damage, but also an improvement in the well-being of peripheral nerves by increasing specific markers such as myelin protein zero (MPZ) and neuregulin 1 (NRG1) [54]. As a result of the same investigation, a dose range of higher efficacy at the nerve target was identified, namely the range of 300–600 mg, which was considered in the product Assonal^®^PEA. Despite some evidence that PEA is an essential mediator of anti-inflammatory, analgesic, and neuroprotective effects in the central and peripheral nervous systems [61], PEA does not possess the direct antioxidant ability to stop the production of free radicals and repair damage to proteins, lipids, and DNA [30]. For this reason, the presence in Assonal^®^PEA of the extract of *Equisetum arvense* L. in combination with PEA80 mesh has already been shown in vitro to support neuronal health by enhancing the antioxidant capacity of PEA thanks to the phenolic compounds, flavonoids and phenolic acids with antioxidant capacity contained in *Equisetum arvense* L. [13,62].

Regarding the reduction of intensity of perceived pain in terms of NPRS and VRS scales after 15 days of constant intake, patients reported a reduction in perceived chronic pain of about 1 point between the two groups compared to T0. The effect was constant even after the dosage change, delineating a time-dependent effect as observed in other studies with PEA supplements combined with potential antioxidants [63,64]. This crucial element might be linked to the reality that PEA, in its micronised or ultra-micronised form, has favourable pharmacokinetic characteristics. It has been shown that, after oral administration, umPEA reaches significant plasma concentrations within a few hours and tends to selectively accumulate in inflamed tissues, contributing to a relatively early therapeutic effect [53]. The effect observed already after 15 days could therefore be attributed to this targeted tissue distribution and the ability of the molecule to act locally to modulate the inflammatory response [65]. Despite what has been demonstrated by Chirchiglia et al., 2018, supplementation with PEA-um at a daily dose of 1200 mg as an adjunct to the classic pharmacological treatment, that is, a non-steroidal anti-inflammatory drug (NSAID), contributed to a statistically significant and time-dependent reduction in pain intensity. In contrast, NSAID alone reduced the severity of pain during attacks but failed to change the intensity of pain during relapses or the number of attacks per month [46,63]. In this study, the effect was significant after 60 days of use [46] by contrast, Assonal^®^PEA showed already after one month to largely limit the intensity of chronic pain. In addition, Assonal^®^PEA also improves the profile of the state of perceived pain in terms of social distress. Despite Chirchiglia et al., 2018 and another clinical study [63,66], Assonal^®^PEA with a reduced PEA dosage of 600 mg promoted optimal results in the treatment of chronic pain, indicating a synergistic role of PEA and *Equisetum arvense* L. maintained on enrolled patients. It also outlined a non-dose-dependent effect of the supplement and confirmed the efficacy of the 600 mg dosage on chronic pain and the optimal results obtained in vitro [54]. At the same time, the patient-reported improvement in perceived pain was also confirmed to be time-dependent on quality of life and overall perception of treatment efficacy using the EQ-5D-5L and GPE scores, respectively.

Concentrating on the components of Assonal^®^PEA’s mechanism of action was made feasible by the clinical investigation. It is known that PEA modulates the endocannabinoid system through the so-called entourage effect, enhancing the action of endocannabinoids such as anandamide (AEA), which act on cannabinoid receptor 1 (CB1) and cannabinoid receptor 2 (CB2). CB1 receptor is expressed mainly in the central and peripheral nervous system, particularly in neurons, including those of the dorsal roots (DRG), and is involved in the modulation of pain transmission [46]. On the other hand, CB2 is expressed predominantly in cells of the immune system; it has also been observed at the level of astrocytes and microglia [67], and its activation improves neuroprotection and anti-nociceptive effect [68,69]. Moreover, PEA intake may be a component of the complex homeostatic system that regulates the basal threshold of inflammation (“modulator of immunoneural homeostasis”) [70,71] and, in turn, the severity and chronicity of perceived pain, given PEA’s capacity to modulate protective responses during inflammation and pain [49,72]. Related to these mechanisms, there is evidence from preclinical studies suggesting that activation of the endocannabinoid system may affect the endogenous opioid system [73]. For example, CB1 receptor activation has been shown to enhance μ-opioid receptor (MOR) signalling, and endocannabinoids may increase endogenous opioid peptide release in certain brain regions involved in pain processing (e.g., nucleus accumbens) [74]. Thus, while PEA does not directly activate opioid receptors, its modulatory role on the endocannabinoid system may result in indirect activation or enhancement of endogenous opioid pathways, contributing to its overall analgesic effect [75]. This possible crosstalk between systems may help explain the observed clinical benefit in chronic musculoskeletal pain.

In addition, the significant analgesic potential of *Equisetum arvense* L. extract has been demonstrated in humans, as shown in another study that found an adjuvant effect when combined with Ibuprofen in treating rheumatoid arthritis [76]. The extract’s rich content of phenolic compounds contributed to its analgesic activity by reducing oxidative stress directly related to inflammation and the pain pathway [77]. Recent research has focused on these biological factors, allowing for the delineation of optimal results on in vitro models that highlight some of the anti-nociceptive actions of *Equisetum arvense* L. in combination with PEA. At the level of the central nervous system, important information was gathered on key chemical pathways, including the endocannabinoid system, Fatty Acid Amide Hydrolase (FAAH), and N-Acylethanolamine acid amidase (NAAA) regulation [78]. Thus, PEA and *Equisetum arvense* L. possess individually documented properties relevant for pain management [59,79]; their mechanisms of action are distinct but complementary, which provides a solid motivation for their combined administration. On the one hand, the anti-inflammatory action previously discussed of PEA and the recognised antioxidant effect of *Equisetum arvense* L., thanks to its bioactive composition of flavonoids, phenolic acids and silica, both lead to the modulation of cyclooxygenase 2 (COX-2) and TNF-α [80].

Although the results obtained suggest a potential benefit of supplementation with Assonal^®^PEA in managing neuropathic pain and improving patients’ quality of life, the present study has certain limitations that must be considered when interpreting the results. Firstly, it is an observational, prospective, non-randomised study without a control or placebo group. This limits the possibility of establishing a direct causal link between the intake of the product and the observed improvements, leaving room for the influence of external factors, such as the placebo effect or the natural course of the disease. Furthermore, the complete absence of dietary evaluation as a possible confounding factor can also represent a possible limitation. However, it should be noted that a distinctive element of this study is the adoption of a before-after design. This methodological choice deserves particular attention as it is strategic in the context of the evaluation of an oral supplement under real conditions of use. To directly observe changes over time within the same individual, this type of design collects data on participants both before (baseline) and after the intervention. In this setting, each participant serves as their own control, so significantly diminishing the influence of inter-individual variability that frequently complicates result interpretation in studies utilising parallel groups. The before-after design also offers the advantage of capturing the dynamics of change over time, allowing not only to measure the effectiveness of the intervention, but also to evaluate its persistence or evolution at different time points. This type of information is crucial in the field of supplements, where effects may be gradual and where it is essential to understand whether any reported benefit is transient or sustained. It should be noted that the main outcomes were measured using subjective self-assessment scales (VRS, NPRS), which, although they are validated instruments and widely used in the clinical field, may be affected by individual variability and psychological influences. Although Assonal^®^PEA itself is not currently marketed worldwide, its components are widely available, and its mechanism of action is consistent with the current scientific understanding of pain modulation and neuroinflammation discussed previously. Therefore, our study should be interpreted not only as a product-specific evaluation but also as a proof of concept supporting the potential synergistic use of PEA and phytochemicals in chronic pain management. Similar to other innovative combinations that emerge initially in tight markets, such investigations could form the basis for future regulatory proposals and wider international application. Future research, conducted with randomised, controlled designs and on larger, homogeneous samples, will be necessary to confirm these preliminary results and to further investigate the efficacy and tolerability profile of this specific formulation. The absence of side effects and 94% responsiveness to Assonal^®^PEA (3 patients only indicated no improvement) in chronic pain represented initial positive data on the analgesic role of the supplement.

## 5. Conclusions

The results of this study indicate that the combination of PEA and *Equisetum arvense* L. (Assonal^®^PEA) effectively reduces chronic pain intensity and improves patients’ quality of life. The significant reduction in NPRS and VRS scores suggests a significant analgesic effect after 15 days of treatment, with further improvements observed up to two months. Additionally, *Equisetum arvense* L. is recognised for its anti-inflammatory and analgesic properties, which contribute to the observed effects. Finally, the reduction of the dose from 2 to 1 tablet per day from the end of T1 did not affect the effectiveness of the treatment, suggesting that a lower dose might be sufficient to maintain the beneficial effects, with potential benefits for patient compliance.

## Figures and Tables

**Table 1:** Anamnestic and demographical characteristics of the study population.

	AA (n = 50)
Age (years)	67.3 ± 10.9
BMI (kg/m^2^)	20.9 ± 2.8
Sex (female/male)	22/28
Smoke (habitual smokers)	3
≥3 alcohol units/day	1

Continuous variables are expressed as means ± standard deviations, categorical variables as counts (percentages), and ratios as x/y. Abbreviations: Active Arm = AA; BMI = Body Mass Index.

## Data Availability

The original contributions presented in this study are included in the article. Further inquiries can be directed to the corresponding author(s).

## Abbreviations

The following abbreviations are used in this manuscript:

ANOVA	Analysis of variance
A.O.U	Azienda Ospedaliero Universitaria (University Hospital Authority)
COX-2	cyclooxygenase 2
DRG	dorsal roots
EQ-5D-5L	European Quality of Life Five Dimension Five Level Scale
GPE	Global Perceived Effect
IL-1β	Interleukin-1 beta
IL-6	Interleukin-6
MOR	μ-opioid receptor
mPEA	Micronised PEA
MPZ	Myelin protein zero
NPRS	Numerical Pain Rating Scale
NPSI	Neuropathic Pain Symptom Inventory
NSAIDs	Nonsteroidal anti-inflammatory drugs
NRG1	Neuregulin 1
PEA	Palmitoylethanolamide
PPI	Pain Intensity Index
SF-MPQ	Short Form McGill Pain Questionnaire
SS	Saints
T0	Timepoint 0
T1	Timepoint 1 (15 days)
T2	Timepoint 2 (one month)
T3	Timepoint 3 (two months)
TEC	Territorial Ethics Committee
TNF-α	Tumor necrosis factor-alpha
umPEA	Ultra-micronised PEA
USA	United States of America
VAS	Visual Analog Scale
VRS	Verbal Rating Scale
